# Out“FOX”ing cancer: the implications of FOXC2 as a putative predictive biomarker for cancer immunotherapy

**DOI:** 10.3389/fmed.2026.1883380

**Published:** 2026-06-30

**Authors:** Kristian M. Hargadon

**Affiliations:** Hargadon Laboratory, Department of Biology, Hampden-Sydney College, Hampden-Sydney, VA, United States

**Keywords:** biomarker, cancer, cancer immunotherapy, FOXC2, tumor immunity

## Abstract

The forkhead box family transcription factor FOXC2 is an important oncogenic driver of several hallmarks of cancer progression. In addition to its well-characterized roles in tumor angiogenesis, metabolic adaptation, and the epithelial-mesenchymal transition that precedes invasion and metastasis, FOXC2 has recently been implicated in tumor immune evasion as well. This Perspective article highlights recent findings concerning the potential value of FOXC2 as a predictive biomarker for cancer immunotherapy and brings attention to data connecting FOXC2 dysregulation to critical determinants of anti-tumor immune outcome, including influences on the makeup of the tumor immune microenvironment and cancer cell responsiveness to type I and type II interferons. In summarizing the current state of knowledge surrounding the immunologic impact of FOXC2 dysregulation in cancer, this article also raises several important questions that will need to be addressed going forward as the field aims to better understand FOXC2′s role in tumor immune evasion so that these insights may be leveraged into translatable interventions that enhance the efficacy of cancer immunotherapy.

## 1 Introduction

Since its discovery in 1993, the forkhead box family transcription factor FOXC2 has emerged as a critical regulator of diverse biological processes across an array of tissues. First recognized as a temporally expressed factor within developing mesoderm and mesenchymal tissue (1), FOXC2 has since been shown to control key spatiotemporal activities in both embryos and adults. Perhaps most widely appreciated for its central role in the early development of the cardiovascular and lymphatic systems (2), FOXC2 indeed exhibits ongoing post-developmental control of these systems through its regulation of angiogenesis and lymphangiogenesis in adults (3, 4). Additionally, beyond these important vascular influences, FOXC2 is now known to govern various aspects of cellular metabolism (5, 6), kidney function (7), wound repair (8, 9), and microbiome composition (10), all of which contribute locally and systemically to normal physiologic function.

Despite the physiologic importance of FOXC2 as a transcriptional regulator, many of the processes it controls are those which often become dysregulated in the context of cancer. Accordingly, FOXC2 has emerged over the last two decades as an important driver of several hallmarks of cancer progression (11). The oncogenic activity of this transcription factor was first described in 2007 by Robert Weinberg's group, who reported elevated expression of FOXC2 in a high percentage of invasive breast carcinomas, particularly those of the basal-like subtype of invasive ductal breast cancer. This report was also the first to demonstrate that FOXC2 promotes cell migration and invasion by supporting the epithelial-mesenchymal transition (EMT) program that is so often a precursor to cancer cell metastasis (12). Since this seminal discovery, overexpression and/or aberrant nuclear localization of FOXC2 have been associated with clinicopathological features of aggressive disease in many cancers of epithelial origin (13–15), with similar findings being reported for some non-epithelial cancers, such as melanoma and glioma, as well (16, 17). During this time, experimental work has demonstrated a role for FOXC2 not only in cell migration and invasion but also several other cancer-promoting processes, including tumor angiogenesis (18), glycolytic and fatty acid metabolism (5, 19, 20), stemness (21), and drug resistance (22–24).

With regard to FOXC2′s specific impact on cancer drug resistance, several studies have reported elevated expression of this transcription factor as a poor prognostic factor for patient response to chemotherapy (13, 25). Mechanistically, FOXC2 has been shown to mediate such resistance by: 1) driving expression of multidrug transport pumps from the ATP-binding cassette (ABC) family of proteins that promote xenobiotic detoxification and efflux (26, 27); and 2) activating pro-survival signaling pathways that prevent drug-induced apoptosis, such as the PI3K-Akt and MAPK pathways (28–30). Additionally, multiple studies have implicated FOXC2 in the oxidative stress response (31–34), leading to speculation that FOXC2 might also confer broad resistance to chemotherapeutic agents whose mechanism of action relies at least in part on their induction of toxic reactive oxygen species within cancer cells. Importantly, though the body of literature describing FOXC2′s impact on patient and cellular responsiveness to various chemotherapeutics is substantial, newly emerging data is now implicating FOXC2 as a potential driver of resistance to cancer immunotherapy as well. Based on the increasing relevance of immune-based interventions to the overall landscape of cancer therapy, this Perspective article aims to bring attention to recent evidence supporting a role for FOXC2 in tumor immune evasion and, in particular, to emphasize the potential significance of FOXC2 as a putative predictive biomarker for cancer immunotherapy going forward.

## 2 A role for FOXC2 in tumor immune evasion—an emerging contributor to cancer progression

Though FOXC2 dysregulation has long been associated with several hallmarks of cancer progression, the oncogenic activity of this transcription factor has only more recently been suggested to play a role in the evasion of anti-tumor immunity. In 2019 our group became the first to report potential immunologic consequences of FOXC2 hyperactivity in cancer, documenting the significance of elevated *FOXC2* gene expression in tumor tissue as a poor prognostic factor for progression-free survival in a small cohort (*n* = 22) of melanoma patients treated with the CTLA-4 checkpoint inhibitor ipilimumab (32). We have since reported a similar negative correlation for progression-free and overall survival in a larger cohort (*n* = 121) of melanoma patients treated with anti-PD1 checkpoint blockade therapy (16). In keeping with previously published studies documenting T cell infiltration of tumors as a predictor of response to immune checkpoint blockade (35–37), we have also found that *FOXC2* gene expression in melanoma tissue samples from the TCGA SKCM cohort (*n* = 480) likewise correlates negatively with CD8+ T cell infiltration, as inferred by each of seven different algorithms commonly used for deconvolution of bulk RNA-sequencing (RNA-seq) data and estimation of immune cell abundance within the tumor microenvironment (TME) (16). Should future experimental studies ultimately validate these bioinformatic observations, it will be interesting to explore whether T cell exclusion from FOXC2-active tumors arises from inhibition of chemokines involved in T cell recruitment to tumor tissue, loss of adhesion molecules that preclude circulating T cell interactions with vasculature in the tumor bed, or vascular/stromal barrier formation that prevents T cell infiltration of tumors.

Although the mechanisms by which FOXC2 activity might interfere with anti-tumor immunity have yet to be thoroughly explored, our work to date highlights interesting possibilities that are worth exploring further in the near future. First, we have recently analyzed multiple RNA-seq datasets from melanoma patient biopsies and performed topology-based Impact Pathway analysis of *FOXC2*-correlated genes to gain insights into the biologic pathways that are predicted to be most significantly impacted by those genes whose expression correlates with that of *FOXC2* (16). Using this approach, we found that the PI3K-Akt pathway was among those predicted to be most significantly impacted by *FOXC2*-correlated genes across multiple datasets, validating in a clinical setting previous experimental work demonstrating FOXC2-dependent activation of this pathway in human cancer cell lines (29, 38). Of note, PI3K-Akt pathway activity in tumors has been associated with a lack of T cell infiltration, as demonstrated by studies investigating T cell exclusion in tumors exhibiting either genomic loss of *PTEN* or genomic amplification/gain-of-function mutations in *PIK3CA* or *PIK3CB* (39, 40). Based on these data and our findings as described above, it is interesting to speculate that FOXC2-associated regulation of genes that control PI3K-Akt pathway activity might also promote T cell exclusion from tumors and confer resistance to immunotherapy through non-genomic, epigenetic influences on this pathway. In this regard, though the precise mechanisms of its epigenetic control remain to be fully elucidated and could involve either chromatin remodeling or recruitment of RNA polymerase—FOXC2 has been found to occupy both enhancer regions and proximal promoter regions near transcription start sites, depending on the target gene (29, 41)—direct FOXC2 gene targets relevant to the PI3K pathway have been reported by multiple investigators and include PDGFRβ, NOTCH1, ITGB1, and stanniocalcin 1 (21, 29, 42, 43), each of which are known to stimulate PI3K-Akt signaling in cancer cells. Future studies will be needed to ascertain whether FOXC2-associated regulation of these specific axes is directly involved in T cell exclusion from tumors, but current data suggest that this transcription factor may indeed serve as a critical hub protein for coordinating activity of a pathway known to be relevant to this process.

In addition to our bioinformatic analyses of *FOXC2*-correlated genes identified from clinical melanoma datasets, we have also gained valuable insight into FOXC2′s oncogenic activity and potential impact on anti-tumor immunity through studies in the B16 murine melanoma model. Specifically, using a CRISPR-Cas9-engineered *Foxc2* knockout variant of the B16-F1 cell line, we found that FOXC2 negatively regulates the expression of several interferon (IFN) pathway genes (32, 44), including the *Ccl5* gene which encodes a chemokine known to be important for recruitment of circulating effector T cells into peripheral tissue (45). In light of these findings, it will be interesting to determine, as highlighted above, whether FOXC2-associated downregulation of CCL5 dampens T cell recruitment to tumor tissue. In addition, beyond serving as another potential mechanism for promoting T cell exclusion from tumors, negative regulation of type I and II IFN pathway genes by FOXC2 in cancer cells also has the potential to confer resistance to effector T cells that do successfully infiltrate tumor tissue, as engagement of these pathways is a well-documented means of mediating anti-tumor immune reactivity and is known to be crucial for the efficacy of cancer immunotherapy (46–50). In this regard, among other IFN pathway genes identified as being negatively regulated by FOXC2 in our murine melanoma model are several downstream effectors of IFN signaling (including genes encoding the STAT1, IRF7, and IRF9 transcription factors) as well as an array of IFN-stimulated genes (multiple members of the OAS, IFIT, and GBP families, for example) that, at least in the context of acute signaling, have been shown to exhibit anti-tumor activity. Also of note, some of the IFN pathway genes downregulated in FOXC2-expressing as compared to FOXC2-deficient B16-F1 melanoma, such as *Irgm1* and *Ifit3*, are known to enhance PD-L1 expression on cancer cells (51, 52). While downregulation of genes that support expression of this inhibitory checkpoint ligand might seem counterintuitive as a pro-tumor function of FOXC2, it is well-established that PD-L1 expression is a strong predictor of response to anti-PD-1 checkpoint blockade, and these data thus suggest a potential mechanism by which FOXC2-overexpressing melanomas might subvert responses to this particular form of immunotherapy. Future studies in preclinical models are needed both to test this hypothesis and to determine whether FOXC2 acts directly or indirectly to regulate expression of IFN pathway genes relevant to tumor immune susceptibility.

Outside of our own work in melanoma, immunologic consequences of *FOXC2* expression in tumor tissue have likewise been reported in the context of other malignancies as well. In one study of esophageal cancer cases from The Cancer Genome Atlas, *FOXC2* was among a group of four high-risk anoikis-related genes whose expression in tumor tissue correlated negatively with immunologic scores and expression of type I/II IFN response genes (53). Though this study found that *FOXC2* expression correlated positively with the presence of M0 and M1 macrophages in tumor tissue, another study recently demonstrated that FOXC2 can also reprogram macrophages toward a pro-metastatic and immunosuppressive phenotype by driving renal tumor cell secretion of LAMA4, which promotes cancer progression and metastatic niche formation through ITGA6-mediated signaling in macrophages (54). Because many tissue/tumor-specific factors ultimately influence myeloid cell plasticity, it is likely that the discrepancy in these studies arises from differences in the overall extracellular milieu in which FOXC2-active cancer cells exist within the esophageal versus renal TME. It remains possible as well that FOXC2 itself might directly regulate macrophage polarization in distinct ways based on intracellular factors that favor either its co-activating or co-repressing functions. In this regard, it is also worth noting that FOXC2 interacts directly with multiple NFAT family transcription factors, which are known to regulate genes with diverse immune-related functions. Interestingly, studies have shown that this interaction can lead to synergistic activation of gene expression as well as FOXC2-mediated suppression of NFAT-driven transcription, depending on which NFAT family member it engages (55, 56). Though these phenomena have not been investigated specifically in the context of cancer, a recent study did show that DNA-facilitated protein-protein interactions between FOXC2 and NFAT1 suppressed the expression of several proinflammatory cytokine- and chemokine-encoding genes known to impact anti-tumor immune function. Specifically, FOXC2 was found to repress NFAT1-driven transcription of IL2, TNF, CXCL5, and CCL2. This suppression was achieved when the DNA-binding domain of FOXC2 was mutated but not when its NFAT interaction domain was, suggesting that FOXC2 can act as a co-repressor of NFAT even at genomic loci that lack consensus FOXC2-binding motifs (56). Going forward, it will be interesting to explore the dynamics of FOXC2-NFAT interactions and how they influence gene expression patterns in cancer cells specifically, as these interactions are indeed likely to influence the recruitment and activity of immune populations that ultimately impact the outcome of cancer immunotherapies. In light of our observation that T cell infiltration of melanomas correlates negatively with *FOXC2* gene expression, it is indeed interesting to speculate that FOXC2-mediated repression of CCL2 and IL-2 in particular might impair T cell recruitment to, and expansion within, tumor tissue.

## 3 Implications of FOXC2 as a predictive biomarker for cancer immunotherapy: from patient stratification to novel combination regimens

Based on its well-documented role as a driver of cancer chemoresistance, FOXC2′s recent emergence as a putative regulator of anti-tumor immunity is intriguing, and early evidence of its predictive utility in small cohorts of melanoma patients undergoing checkpoint blockade immunotherapy warrants further investigation in larger cohorts spanning diverse cancer types and immunologic interventions. Importantly, though it has not been explored elsewhere in the context of immunotherapy, *FOXC2* gene expression in tumor tissue has previously been found to predict patient response to a range of chemotherapeutics across diverse cancer types (13). While it should be emphasized that the bulk RNA-seq data used for these analyses cannot distinguish the cellular source of gene expression nor predict the actual level or localization of FOXC2 protein in the cells within a tumor, it is interesting that even this most basic readout of *FOXC2* gene activity carries such broad predictive value. The significance of FOXC2 as a prognostic biomarker has also been validated by several other groups using more refined immunohistochemical analysis of tumor biopsy specimens, with many demonstrating that elevated FOXC2 protein level and nuclear localization specifically within cancer cells are correlates of aggressive clinicopathological features of disease and poor patient survival (11, 57–63). Based on this precedent, establishing FOXC2 activity, either through gene expression profiling or immunohistochemical analysis of biopsy specimens, as a predictive biomarker for response to cancer immunotherapy has promising application, at the very least, for patient stratification during therapeutic planning. Indeed, insight into which patients are expected to respond best to a given immunotherapy would inform front-line therapy decisions and preclude those less likely of responding from experiencing the deleterious immune-related adverse events that often accompany immune-based interventions, particularly checkpoint blockade regimens that act broadly on T lymphocytes regardless of their specificity for tumor antigen (64). Likewise, in light of our preliminary data implicating a role for FOXC2 in T cell exclusion from tumor tissue, insight into FOXC2 activity within tumors might also be particularly informative for identifying patients with potential to respond to natural T cell or CAR-T-based adoptive cell transfer therapies, where successful infiltration and retention within solid tumors remains a significant challenge to therapeutic efficacy.

In addition to its potential utility for patient stratification, information about FOXC2 activity might also effectively be coupled with other more well-established biomarkers of response to immunotherapy, such as PD-L1 expression, immune infiltration, tumor mutational burden, and microsatellite instability (MSI) status, to inform novel combination regimens that might better support patient responses to immunotherapy. For example, patients whose tumors display indicators of poor response to checkpoint blockade—such as an immunologically “cold”, non-inflamed microenvironment—but which otherwise do not exhibit substantial FOXC2 activity might benefit from interventions that ultimately support checkpoint blockade therapy, such as cancer vaccine regimens that elicit tumor antigen-specific T cell responses or chemotherapy regimens that promote immunogenic tumor cell stress/death and thereby yield a more immunologically favorable TME (65). Likewise, tumors without substantial FOXC2 activity might be more readily infiltrated by adoptively transferred T or CAR-T cells that could also be supported by checkpoint blockade.

Another exciting possibility for intervention against FOXC2-active tumors that might extend the reach of cancer immunotherapy is the inhibition of either FOXC2 itself or gene products/pathways transcriptionally activated by FOXC2. Although transcription factors have historically been considered “undruggable” targets, Castaneda et. al. have described MC-1-F2 as a novel pharmacologic inhibitor that prevents FOXC2 nuclear translocation and targets the transcription factor for proteasomal degradation (66, 67). This inhibitor has been shown to reverse the EMT phenotype in both breast and prostate cancer cell lines, and it also increases prostate cancer cell sensitivity to docetaxel, a chemotherapeutic known to promote immunogenic cell stress via its induction of calreticulin translocation to the cell surface (68). Though MC-1-F2 displays physiochemical properties that would likely preclude its development for clinical use, several more favorable analogs of this inhibitor have also been identified and may prove useful as therapeutic agents in the future (69). Based on the biologic effects of MC-1-F2 observed to date, treatment of FOXC2-hyperactive cancers with a more favorable analog that exhibits similar anti-tumor effects might indeed be capable of restoring tumor immunogenicity and countering FOXC2-associated resistance to immunotherapy. Of course, before such combinatorial interventions can be pursued, it will be imperative to evaluate *in vivo* the pharmacokinetic/pharmacodynamic profile of any FOXC2 inhibitor under investigation in order to assess potential toxicities, particularly in light of FOXC2′s normal physiologic functions in the vascular, lymphatic, and renal systems. Should such inhibitors prove too toxic for systemic administration, it might also be worthwhile exploring RNAi-based gene therapy strategies to suppress FOXC2 more specifically in cancer cells using, for instance, vectors that have been engineered to express FOXC2-targeting shRNA under the control of a tissue-specific promoter. Tissue-specific silencing has already been successfully achieved against other genes in therapeutic settings for cancer as well as chronic virus infection (70, 71), and such an approach has the potential to offset the oncogenic functions of FOXC2 in cancer cells while preserving the transcription factor's normal functions in non-cancerous tissue. Alternatively, should FOXC2 itself prove to be too challenging to target directly, selective targeting of other FOXC2-regulated gene products that are active in oncogenic pathways known to promote tumor immune evasion may enhance the immune susceptibility of FOXC2-overexpressing cancers. In this regard, already-approved drugs that inhibit immune subversive pathways stimulated by FOXC2, such as the PI3K-Akt and MAPK pathways, might also prove useful as therapeutic adjuncts to immune-based interventions. Such inhibitors have already been shown to increase the sensitivity of tumor cells to platinum-based chemotherapeutics in FOXC2-overexpressing cancers (24, 72), and restoring susceptibility to these known inducers of immunogenic cell death may indeed support natural anti-tumor immune responses as well as those elicited via immunotherapy.

## 4 Conclusions and future considerations

Over the last several years oncogenic dysregulation of the FOXC2 transcription factor has become increasingly appreciated as an important driver of cancer progression. In addition to its regulatory influence over several core hallmarks of cancer, FOXC2 confers broad resistance to a range of widely used chemotherapeutic agents, underscoring the clinical relevance of this transcription factor as a determinant of patient outcome. In addition to these well-established oncogenic consequences of FOXC2 hyperactivity, it has only recently become apparent that FOXC2 also exhibits immunologic influences relevant to cancer progression, with data emerging that the transcription factor plays a role in shaping the cellular makeup of the TME as well as cancer cell responsiveness to type I and type II IFN. Our own analyses of small melanoma patient cohorts indeed indicate that FOXC2 may have utility as a predictive biomarker for patient response to checkpoint blockade immunotherapy. Importantly, with preclinical studies having demonstrated the anti-tumor potential of selectively targeting this transcription factor in FOXC2-dysregulated cancers, there may exist previously untapped opportunities to enhance tumor immune reactivity through combinatorial interventions that couple immunotherapy with FOXC2-targeting regimens.

In highlighting data relevant to FOXC2′s immunologic influences and their potential implications for the treatment of cancer, it is important to consider as well the questions raised by these early findings and the future work that is necessary to solidify their impact on disease management going forward. First, it will be necessary to validate our preliminary observations of the predictive value of *FOXC2* gene expression as a biomarker for melanoma patient response to checkpoint blockade therapy in significantly larger patient cohorts. Determining whether FOXC2 protein expression/subcellular localization might have similar predictive utility in such settings will also provide new insight into FOXC2′s potential value as a biomarker. It is also important to acknowledge that the majority of data that to date support immune-subversive functions of FOXC2 come from studies in melanoma, and future work will need to be extended to other cancer types to determine whether FOXC2 might have utility as a pan-cancer immunotherapy biomarker. Considering the variability in immune microenvironments across specific tissues (with immunogenic neoantigen release in MSI-high colorectal cancer, highly immunosuppressive stroma in pancreatic cancer, liver-specific immune tolerance in hepatocellular carcinoma, etc.), validation of FOXC2′s role in response to immunotherapy across distinct cancers is critical. As well, future studies should also take into account whether/how the predictive value of FOXC2 as a biomarker applies across diverse patient demographics and immunotherapeutic modalities.

Although work to date has evaluated *FOXC2* gene expression as an isolated predictor of response to checkpoint blockade therapy, there remains a need to integrate this information with more clinically-established biomarkers of response to immunotherapy in order to determine whether FOXC2 acts independently or in concert with other determinants to influence patient outcome. Future studies should therefore assess how *FOXC2* expression predicts the response to tumors with high versus low PD-L1 tumor proportion scores, high versus low tumor mutational burden, and T cell-inflamed versus non-inflamed signatures. As there still exists a range of outcomes among responders identified by each of these validated biomarkers, it is interesting to speculate that FOXC2 activity might further refine clinical predictions and thus add value as part of a multi-panel biomarker that incorporates this information with other well-characterized parameters known to influence patient outcome.

Importantly, although analyses of clinical datasets have suggested a role for FOXC2 in T cell exclusion from tumor tissue and implicated the transcription factor in the activation of immune-subverting pathways, to date experimental work on FOXC2′s regulation of anti-tumor immunity is largely lacking and represents a major void in the field that future studies will need to fill. Does FOXC2 activity in cancer cells actively limit tumor infiltration by T lymphocytes, and if so, how? Do FOXC2′s pro-survival functions preclude sufficient release of tumor antigen for initiation of the cancer-immunity cycle? Might FOXC2′s negative regulation of cytokine/chemokine expression limit the recruitment of T cells into tumor tissue even if an anti-tumor response is elicited? Does FOXC2-mediated repression of IFN pathway genes in cancer cells subvert the anti-tumor activity of immune effectors that do infiltrate tumor tissue? What factor (s) ultimately dictate whether FOXC2 acts as a co-activator or co-repressor of NFAT family members, and how might FOXC2-associated regulation of NFAT target genes impact the outcome of an anti-tumor immune response?

Finally, while FOXC2 holds great promise as a predictive biomarker that may optimize therapeutic decision-making at the time of a cancer diagnosis, what is perhaps most attractive about acquiring insight into FOXC2 activity in tumor tissue is the potential that information may hold for targeted intervention against FOXC2. Significant work remains to be pursued in this regard, but pharmacologic inhibition of FOXC2 has already shown pronounced anti-cancer effects in *in vitro* settings. How these results will translate to *in vivo* settings, where FOXC2 serves important functions in non-cancerous tissue, remains to be investigated and will need to be carefully studied going forward. Should work in this area reveal FOXC2 to be a logistically actionable target, though, it will be exciting to explore how inhibition of this transcription factor, either pharmacologically or through tissue-specific gene silencing, might be used to complement immunotherapy as part of combination regimens. And as noted above, should FOXC2 itself prove undruggable, it will be worth exploring whether other FOXC2 target genes might instead be inhibited in FOXC2-hyperactive cancers in ways that ultimately support immunotherapy. Collectively, addressing these issues and the remaining questions raised herein will greatly improve our understanding of FOXC2 and its oncogenic functions, and these new insights may indeed allow us to ultimately out“FOX” cancer in a way that significantly extends the reach of immunotherapy in the future.

## Data Availability

The original contributions presented in the study are included in the article/supplementary material, further inquiries can be directed to the corresponding author.
